# Electrodeposited Heusler Alloys-Based Nanowires for Shape Memory and Magnetocaloric Applications

**DOI:** 10.3390/ma17020407

**Published:** 2024-01-13

**Authors:** Michal Varga, Ladislav Galdun, Marek Vronka, Pavel Diko, Oleg Heczko, Rastislav Varga

**Affiliations:** 1 Faculty of Materials, Metallurgy and Recycling, Technical University of Kosice, Letna 9, 040 01 Kosice, Slovakia; varga@tuke.sk; 2 Centre for Progressive Materials, Technology, and Innovation Park, Pavol Jozef Safarik University in Kosice, Tr. SNP 1, 040 11 Kosice, Slovakia; ladislav.galdun@upjs.sk; 3 FZU—Institute of Physics of the Czech Academy of Sciences, Na Slovance 1999/2, 182 00 Prague, Czech Republic; vronka@fzu.cz (M.V.);; 4 Institute of Experimental Physics, Slovak Academy of Sciences, Watsonova 47, 040 01 Kosice, Slovakia; dikos@saske.sk

**Keywords:** Heusler alloy, nanowires, electrodeposition, shape memory, magnetocaloric effect

## Abstract

In this article, the downsizing of functional Heusler alloys is discussed, focusing on the published results dealing with Heusler alloy nanowires. The theoretical information inspired the fabrication of novel nanowires that are presented in the results section of the article. Three novel nanowires were fabricated with the compositions of Ni_66_Fe_21_Ga_13_, Ni_58_Fe_28_In_14_, and Ni_50_Fe_31_Sn_19_. The Ni_66_Fe_21_Ga_13_ nanowires were fabricated, aiming to improve the stoichiometry of previous functional Ni-Fe-Ga Heusler nanomaterials with a functional behavior above room temperature. They exhibit a phase transition at the temperature of ≈375 K, which results in a magnetocaloric response of |Δ*S_M_*| ≈ 0.12 J·kg^−1^·K^−1^ at the magnetic field change of only *μ*_0_Δ*H* = 1 T. Novel Heusler alloy Ni_58_Fe_28_In_14_ nanowires, as well as Ni_50_Fe_31_Sn_19_ nanowires, are analyzed for the first time, and their magnetic properties are discussed, introducing a simple electrochemical approach for the fabrication of nanodimensional alloys from mutually immiscible metals.

## 1. Introduction

Heusler alloys (HA) are materials with the stoichiometric composition of X_2_YZ. They became well-known due to their peculiar properties, which include multiple functionalities, such as superconductivity, thermoelectricity, half-metallicity or shape memory, and magnetocaloric or multi-caloric behavior. Heusler alloys crystallize into an ordered *L2*_1_ structure (space group *Fm-3m*). A sub-group of Heusler alloys with the composition of XYZ is called Half-Heusler alloys. The members of this group are often found in thermoelectric or half-metallic functional materials. They crystallize in the *F-43m* space group of the *C1_b_* structure [1]. It is shown that the mentioned functionalities, together with the overall behavior of the Heusler alloy materials, depend on their structure and composition [1]. All of the mentioned functionalities can be easily tuned, aiming to improve or choose the temperature and magnitude of the desired effect. The tuning is based on a simple composition or stoichiometry change. The changes result in substitution, doping, or concentration changes [2,3,4]. Substitution and doping often result in a disorder of the original cubic structure of the Heusler alloys. The disordered structures remain cubic, but the positions of the individual atoms interchange, resulting in a lower symmetry of the resulting structure. The most often observed disordered structures are *B2*, *A2*, or *D0*_3_, with the space groups *Pm-3m*, *Im-3m*, and *Fm-3m*, respectively [1]. On the other hand, tuning the Heusler alloys with dopants often maintains the ordered *L2*_1_ HA structure. This is achieved by a compatibility of the dopants with the atomic ordering of the original constituents.

The division of Heusler alloys based on their functionality was mentioned earlier. Superconductive Heusler alloys offer multiple functional candidates [5,6,7]. Spin polarization was achieved among Heusler alloys based on the Co_2_YZ composition [8,9,10]. One of the most applicable Heusler alloys’ functionality is the capability of a martensitic transformation. Martensitic transformation is a composition-dependent feature of several Heusler alloys. It is a diffusionless phase transformation between a high-symmetry, high-temperature austenitic phase and a low-symmetry, low-temperature martensite [11]. Martensitic transformation in Heusler alloys is manifested through structural changes, which result in functionalities like the shape-memory effect—reversible changes of the material’s shape and magnetocaloric effect, where changes of magnetic ordering and change of the material’s overall entropy lead to temperature alterations in the alloys [12].

### 1.1. Downsizing of Heusler Alloys

In order to further expand the application possibilities of HA materials, it is possible to choose a path towards downsizing, which allows further functionality when nanoscopic dimensions are achieved. It is well known that a bulk material becomes a nanomaterial when its properties change abruptly at a certain scale. Downsizing of HAs became popular after the discovery of half-metallicity in HAs by de Groot et al. [13,14,15,16]. The preparation was first started with the fabrication of thin HA films that showed half-metallic behavior and high values of spin polarization [17]. HA films can be fabricated using various physical or chemical approaches. These include direct current or radio-frequency magnetron sputtering, molecular beam epitaxy, physical layer deposition, laser beam epitaxy, plasma sputtering, or even electrodeposition [18].

Analysis of downsized magnetic materials shows various size-dependent changes within the magnetic nature of the prepared material. One of the most significant effects of HAs downsizing can be seen in their coercivity behavior [19,20], which changes due to the larger surface area of a nanostructured HA. A larger surface area promotes a more efficient temperature exchange, and the coercivity of a magnetic nanomaterial shows a high temperature dependence.

A different key parameter in the resulting HA film properties is the used substrate. The substrate introduces constraints to the fabricated HA films, with pressures of up to units of MPa. Such an effect may result in an alternating size of the magnetic domains, which alters the magnetic properties [18]. One of the first downsized HA films was produced in 1982 during the research on the effect of annealing on the magnetic properties of HAs Cu_2_MnIn, Cu_2_MnAl, and Cu_2_MnSn [21]. These films had the lowest thickness down to 1 μm. True nanodimensionality of the HA films was achieved around the year 1990 when one of the first nanodimensional HA films was produced. Its composition was Pt-Mn-Sb and it was fabricated from subsequent layers of the individual metals of Pt, Mn, and Sb, which were later annealed to form the *L2*_1_ cubic structure of HAs [22].

### 1.2. Heusler Alloy Nanoparticles

HAs are also produced in the form of nanoparticles. The production methods vary from template-assisted growth in SBA-15 substrates or bottom-up vapor deposition to a top-down ball milling approach, which is unsuitable for shape-memory nanoparticles due to the introduction of impurities, agglomeration, or minor phases and defects into the system [23]. Vapor-deposition methods seem suitable mostly for binary systems, such as Fe_3_Si HA nanoparticles with the *D0*_3_ structure, although chemical vapor deposition (CVD) can be used to obtain stable HA phases after the chemical reaction on the substrate surface [23]. Research on HA nanoparticles shows that the functionality of multiple HAs is preserved at the nanoscale. The size effect is mostly associated with the change of the magnetic properties, such as a typical increase of the coercive field with decreasing particle size [23]. Moreover, coercivity dependence may be ascribed to a transition between the nanoscale’s multi-domain and single-domain system.

Although the functional downsized HAs occur predominantly in the form of thin films, multiple shape-memory HAs have been prepared in the form of nanoparticles. HA nanoparticles were prepared from Co_2_FeGa HA, and the *L2*_1_ cubic phase was confirmed by the extended X-ray absorption fine structure (EXAFS) method [24]. Cu-Mn-Al HA nanoparticles showed a diameter from 4 to 90 nm [25]. Replacing the Mn with Fe resulted in Co_2_FeAl nanoparticles, which crystallized in an *A2*-disordered structure and showed size-dependent properties, such as decreasing coercivity and soft magnetic behavior compared to the bulk material [19]. A different set of Co_2_FeAl nanoparticles was prepared by reduction of precursor chemicals under H_2_ gas, resulting in a *B2* structure and aiming for half-metallic HA nanoparticles [26]. Pulsed laser deposition was used to prepare superparamagnetic Fe_2_CrAl nanoparticles [27]. The high-energy ball milling method was uniquely successful in the preparation of Ni-Mn-In HA nanoparticles for magnetocaloric applications in the temperature range of 270 to 310 K [28]. The nanoparticles showed a more favorable magnetocaloric response compared to the bulk materials, mainly due to a lower hysteresis, a 17% increase in the magnetocaloric response and a higher accuracy of the indirect magnetic entropy estimation (in relation to the direct measurement) compared to the bulk alloy [28]. Ni_51_Mn_33.4_In_15.6_ nanoparticles were also prepared by ball milling, and they showed increasing magnetization with decreasing size and lower *H_C_* values [20]. A different set of Ni-Mn-In nanoparticles was prepared using laser ablation. These showed a diameter of 28 nm, together with a formation of somewhat bigger clusters. Nevertheless, this set of Ni-Mn-In nanoparticles shows the capability of magnetic field-induces strain (MFIS) above 250 K [29]. Ball milling was also used to fabricate Ni-Mn-Ga nanoparticles that showed small crystalline sizes and a size-dependent martensitic transformation capability, which was not observed below the diameter of 50 nm [30]. Shape-memory effect tuning for high-temperature shape-memory applications was also discussed during the research of Co-Ni-Ga nanoparticles, which showed a 30 to 80 nm diameter [31].

### 1.3. Heusler Alloy Nanowires

New shapes of the prepared nanomaterials are being investigated to increase the functionality and applicational possibilities of the downsized HAs. A cylindrical wire shape brings additional functionality into the system due to a well-defined and strong shape anisotropy [7]. Chronologically, the first HA nanowires were prepared in 2010 from a binary Fe_3_Si HA. This research used a diffusion-driven crystal structure transformation to fabricate monocrystalline Fe_3_Si nanowires [32]. HA nanowires have been further studied since 2012 when the first ternary HA nanowires were prepared by electrospinning [33]. Using this approach, Co_2_FeAl nanowires have been prepared [33]. The nanowires were prepared using electrospinning from nitrates of the constituent metals together with polyvinyl alcohol and polyvinylpyrrolidone, which were added to the solution to control the viscosity and, subsequently, the nanowire diameter [33]. The resulting fibers were annealed after electrospinning to remove the polymers, yielding the HA nanowires [33]. The nanowires showed a polycrystalline *B2*-, together with the *A2*-disordered HA structures and a granular morphology (grain size between 10 and 40 nm) [33]. They showed a ferromagnetic behavior with a high Curie temperature, and their diameter varies from 50 to 500 nm [33]. Moreover, the first Co_2_FeAl nanowires showed an unusual increase of magnetization above 600 K at a low field, so differential scanning calorimetry was used to study the presence of the phase transformation, which was not confirmed [33]. On the other hand, the magnetization increase was not present at the high field measurement. Therefore, it was ascribed to the granular nature of the fabricated nanowires and misalignment of the magnetization vectors of the individual grains at the low field *M*(*T*) dependence [33].

HA nanowires with the composition of Ni_2_MnGa have also been prepared using the electrospinning method at 15 to 20 kV from nitrates of the individual metals [34]. After the electrospinning, the fibers undergo calcination to remove the polymers and transform the nitrides into oxides, which is followed by thermal annealing, resulting in the Ni_2_MnGa HA [34]. This alloy shows unique magnetocaloric properties, and the nanowires show relatively high values of the magnetic entropy change due to the second-order phase transformation [34]. Moreover, the Ni_2_MnGa nanowires prepared by electrospinning exhibit a broad working temperature region of up to 150 K [34]. On the other hand, the occurrence of minor nitride phases, as well as antiferromagnetic oxides, indicates a need for further optimization of the electrospinning process of HA nanowires. The electrospinning method is robust and controllable, offering the possibility to prepare a large amount of nanowires [34]. Nevertheless, the nanowires’ post-production treatment and random alignment might render the nanowires unsuitable for applications under an applied magnetic field, contrary to the utilization of shape anisotropy of isolated nanowires.

HA nanowires can also be fabricated using a template. By now, several templates have been used. The first trials were performed using a mesoporous silica template—SBA-15 [35]. This template was used to prepare Co_2_FeGa nanowires, which had a length of 1 μm and a diameter of 50 nm [35]. The nanowires showed a core-shell structure with the Co_2_FeGa single crystalline *L2*_1_ core and outer shell from a Co-magnetite [35]. Electron holography on a single Co_2_FeGa nanowire revealed dipole-like stray fields with internal magnetic induction of up to 1.15 T and approx. 10 nm non-magnetic surface layer [35]. The results were confirmed by electron energy loss spectroscopy as well as XRD and EXAFS methods with up to 5 nm of lateral resolution [35]. SBA-15 was later employed during the fabrication of another set of Co_2_FeGa nanowires [36]. These nanowires showed small diameters of up to 7 nm and the length of 125 to 200 nm [36].

Another promising method of nanowire fabrication can be found in electrochemistry. In order to prepare metallic nanowires using the electrochemical approach, a template has to be used. The template can be any nanoporous material capable of incorporating an electrical contact with the electrochemical bath to transmit electrical current. One of the most promising templates is the Anodized Aluminum Oxide (AAO) membrane, which is built of highly-ordered nanodimensional pores into which the nanowires can be grown [37]. Recently, AAO membranes were used in combination with magnetron sputtering to fabricate multiple HA nanowire materials [38].

In 1953, Keller et al. studied anodic oxide coatings on aluminum metal surfaces [39]. At the time, the anodization of aluminum was widely used as a form of passivation of the metal’s surface [40]. Later, it was found that the anodic oxide coating forms in two major ways [41]. The first forms a continuous surface at the free active spaces on the surface of the aluminum metal. The second mechanism, however, results in a surface covered with ordered pores. The pores are created when the Al^3+^ ions that originate during the anodization process escape into the solution and leave a porous structure at the original surface of the pure aluminum. The created pores on the anodized surface then grow in depth when the anodization process continues [42]. The physical model and electrochemistry behind the process have been numerously described in the previously published work [37,43]. The growth of the formed pores was later studied, and it was shown that it is possible to govern the pores’ formation and dimensions. Followingly, a new electrochemical electrode for analysis of the chemical solution composition was prepared using porous aluminum oxide [44]. These results have been published in the year 1987 [44], but they were quite inconclusive. Nevertheless, the group decided to employ a porous membrane for the growth of metallic (platinum) nanowires from a solution of metallic salts and ligands using the electrochemical approach [44].

**Figure 1:**
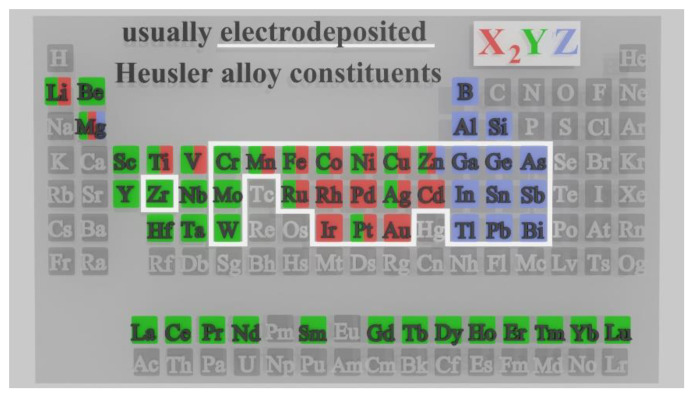
Periodic table of elements, showing the constituents of Heusler alloys with the highlighted portion of metals that can be fabricated by electrodeposition. Information in the Figure was obtained from references [1,45].

Electrodeposition is an electrochemical process during which a metallic monolayer or multilayer is created on a conducting surface (working electrode). The new material layer is created based on an electrochemical reaction (reduction) that occurs between the working electrode and the electrolyte within the electrochemical cell. The elements that are usually electrodeposited from simple salts are shown in Figure 1 [45], in connection with the elements that represent the usual constituents of Heusler compounds [1].

It was shown numerous times that it is also possible to use the electrochemical approach to prepare materials that contain more than a single element within their resulting composition. At first, the published research focused on the preparation of single-element nanowires [46] or nanodimensional disks [47]. Later, binary alloy nanowires were prepared [48]. Some of the most interesting binary nanowires for HA research were the Permalloy, Galfenol, or NiGa nanowires [49,50,51]. In this research, the possibility of co-electrodeposition of iron-gallium, nickel-iron, and other HA constituents was shown, enabling the electrodeposition of ternary HA nanomaterials.

The first article to study electrodeposited ternary HA nanowires employed a potentiostatic direct-current electrodeposition of Co-Ni-Ga nanowires into the AAO membrane [52]. Different potentials were applied to fabricate multiple samples with different compositions from electrochemical solutions of sulfides of the constituent metals and additives [52]. The highest achieved gallium content was 17 at.% [52]. The nanowires had a diameter of 200 nm and showed a polycrystalline *fcc* structure [52]. Moreover, their coercivity decreased with temperature, which was ascribed to a thermal activation mode. The nanowires showed two contributions to the overall anisotropy, which comprised the magnetostatic interaction among the neighboring nanowires together with the shape anisotropy of the individual nanowires [52].

Later, alternating-current (AC) electrodeposition was employed to prepare nanowires with the chemical composition of Fe_2_CoSn. The Fe_2_CoSn nanowires showed the *A2* disorder together with the ordered *L2*_1_ HA structure [53]. Their diameter was 40 to 60 nm with the grain size of 35 nm. During this research, various deposition potentials were used for electrodeposition to study the magnetic and transport properties of the resulting nanowires and their suitability for spintronic devices.

AC electrodeposition was also used to fabricate the first quaternary HA nanowires with the composition of Co_2_Mn_0.5_Fe_0.5_Sn [54]. These nanowires also showed an ordered *L2*_1_ HA structure, accompanied by the *B2* and *A2* disordered cubic phases [54]. The quaternary nanowires had a diameter of 60 nm and showed 100% spin polarization, making them suitable for spintronic research and applications [54]. Moreover, voltage variations were also introduced during the nanowires’ production, which resulted in the deposition of potential-dependent coercivity and saturation magnetization of the quaternary Co-Mn-Fe-Sn nanowires [54].

Further, HA nanowires include the Co_2_FeIn nanowires prepared by DC electrodeposition with a diameter of ≈180 nm [43]. The Co_2_FeIn nanowires crystallize in an *A2*-disordered Heusler phase and show magnetic features suitable for spintronic devices or magnetic racetrack memory [43]. Nanowires with diameters of 30 and 60 nm with the composition of Co_2_FeSn were also prepared by DC electrodeposition [55]. These showed a *B2* disorder that was confirmed by XRD and electron diffraction [55]. Interestingly, these nanowires showed an easy axis perpendicular to the individual nanowires’ longitudinal axes [55]. The magnetization process is presumed to exhibit a coherent rotation of the magnetization vectors due to the neighboring nanowires’ competing shape anisotropy and magnetostatic interaction [55]. Another set of Co_2_FeSn nanowires with a diameter of 50 nm was fabricated by pulsed electrodeposition [56]. Despite a *B2* disorder, these nanowires exhibit 100% spin polarization, making them ideal for spintronic applications [56].

Nanowires with the chemical composition of Co_2_MnSn were prepared for the first time by an AC electrodeposition using sinusoidal waveforms, and they crystallized into an *A2*-disordered HA structure [57]. It was shown that using the amplitude of 16 V and 17 V results in the final product of nanowires with the X_2_YZ HA stoichiometry [57]. This research also showed that the Co_2_MnSn nanowires exhibit a strong shape anisotropy, with a magnetocrystalline contribution, that results in the easy axis of the nanowires being parallel to the longitudinal nanowires’ axes [57]. Angular dependence of the magnetization behavior revealed that the magnetization process shows vortex domain walls at low angles and transversal domain walls at the high angles of the applied magnetic field [57]. Fabrication of the Co_2_MnSn nanowires also unveiled a current density distribution in the electrolyte, showing constant values of the current density inside the individual pores of the AAO membrane with a sharp current density increase after the nanowires overgrow the pore length [57].

Further research on Co-based nanowires studied the influence of electrodeposition parameters on the structural and magnetic properties of Co_2_MnAl nanowires inside the AAO template [58]. XRD and electron diffraction revealed the presence of *B2* disorder. The crystallinity of the electrodeposited nanowires improved with the increasing pH value of the electrolyte up to the value of pH = 3 [58]. Analysis of the magnetic properties revealed a high uniaxial anisotropy and squareness (*M_R_*/*M_S_*) and increasing *M_S_*, *M_R_*, and coercivity of the fabricated nanowires with higher pH of the electrolyte [58]. The article also mentions the suitability of electrodeposited 1D nanowires for miniaturized devices and the applicability of the nanowires for spintronics due to a high Curie point [58].

Around the year 2020, nanowires from shape-memory HA were published for the first time. HA nanowires with an off-stoichiometric Ni-Mn-Ga chemical composition that have been prepared by DC electrodeposition show a diameter of approx. 200 nm and a cubic crystal structure [59]. The article highlights the suitability of shape-memory nanowires in sensing and actuation due to a faster response of the shape-memory nanomaterial compared to piezoelectric or magnetostrictive devices [59]. Moreover, linear size changes might be achieved with a magnetic field upon the fabrication of nanowires with a magnetic field-induced strain [59]. The outcomes of the article discuss the increase of the Curie point and martensitic transformation temperature with Ni content and the decrease of the Curie point with higher Mn content (probably due to the antiferromagnetic Mn-Mn interactions) [59].

Electrodeposited nanowires can also show large magnetoresistance, as was shown for the first time by Wei et al. on electrodeposited Co_2_FeGa nanowires with diameters of 30, 60, and 110 nm [60]. Recently, nanowires with the HA composition of Ni-Fe-Ga were published for the first time. In this pioneering research, the stoichiometric nanowires and nanotubes showed application possibilities in high-frequency devices despite the lack of a functional shape-memory behavior [61].

A summary of the published results dealing with HA nanowires up to date can be found in Table 1.

Table 1 shows that the research on HA nanowires is an unexplored field. The table summarized how the research on HA nanoarchitectures progressed since the first fabricated binary HA nanowires. It shows that HA nanowires exhibit promising application-ready behavior in various spheres of research from spintronic to shape-memory applications. Due to the low number of articles that currently report on HA nanowires, we decided to further explore the field through the fabrication of well-known HAs in the form of nanowires [64,65] as well as the fabrication of novel HA materials within this article.

**Figure 2:**
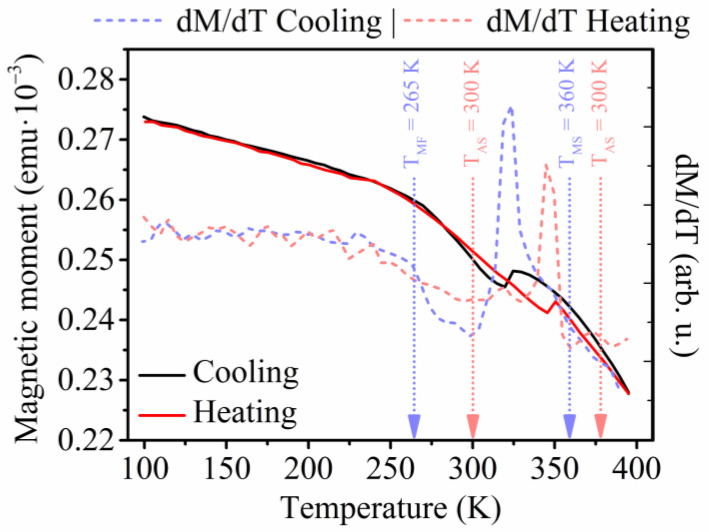
Low field (100 Oe) temperature dependence of magnetization of our first set of Ni-Fe-Ga nanowires that showed martensitic transformation behavior (adapted from Ref. [60]).

A recent article by our group studied off-stoichiometric nanowires with martensitic transformation behavior (Figure 2) that was studied by First Order Reversal Curve (FORC) and Temperature First Order Reversal Curve (T-FORC) analysis [64].

Our following research focused on the electrodeposition of Ni-Mn-Ga HA nanowires from an optimized electrodeposition bath to confirm the hypothesis of the parallel alignment of the nanowires being responsible for a higher magnetic entropy change compared to randomly aligned nanowires (Δ*S_M_* ≈ 0.15 J·kg^−1^·K^−1^ at the higher applied magnetic field change of 2 T) [65]. These results confirmed the nanowires’ parallel alignment suitability for practical applications. The approach to obtain the desired chemical composition within our previous research was similar to the previous Ni_2_FeGa nanowire samples (optimization of the electrodeposition approach), and the optimized electrodeposition bath consisted of 325 mM NiSO_4_, 300 mM MnSO_4_, 28 mM Ga_2_(SO_4_)_3_, 40 mM Na_3_-Citrate, 315 mM H_3_BO_3_, with pH = 4.1. The deposition took place for 6 minutes and the current density was optimized for 100 mA·cm^−2^ [65].

The composition of the nanowires was determined from an SEM-EDS analysis. The resulting average composition taken from various spots from the membrane that contained the nanowires was Ni_65_Mn_20_Ga_15_ [65].

The XRD analysis revealed the presence of a cubic HA phase together with a martensitic structure at room temperature [65]. The structural analysis corresponded to the magnetization measurement, which confirmed first-order phase transition behavior at the temperature of ≈350 K [65]. Various approaches confirmed the behavior by correlating the structural, magnetic, and magnetocaloric analysis of the electrodeposited Ni-Mn-Ga nanowires [65]. The broad magnetization hysteresis of the Ni_65_Mn_20_Ga_15_ nanowires was ascribed to an inhomogeneity of the nanowires’ composition since the shape-memory properties of the Ni_2_MnGa HA change with the alloy’s composition [65].

**Figure 3:**
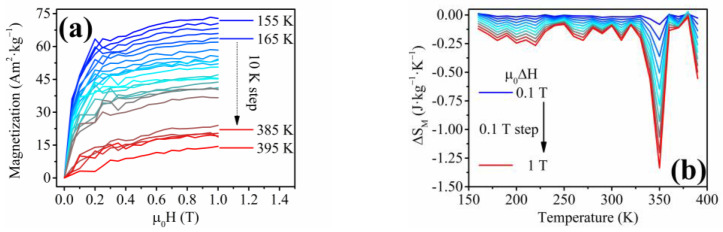
(**a**) Isothermal magnetization measurements of the prepared Ni_65_Mn_20_Ga_15_ nanowires showing a large gap around the temperature of 350 K, which can be ascribed to a first-order phase transformation; (**b**) Magnetic entropy change of the fabricated nanowires with the maximum entropy change of Δ*S_M_* ≈ −1.3 J·kg^−1^·K^−1^ at the temperature of 350 K and the magnetic field change of 1 T (reprinted with permission from [65]).

A decrease of the saturation magnetization with temperature was observed within the isothermal measurements (Figure 3a), together with a slight shifting of the saturation magnetic field into lower values. The first-order phase transition behavior was apparent, appearing as a large gap between the individual measurements of magnetization at different temperatures [65].

The peak values of the magnetic entropy change exceeded Δ*S_M_* ≈ −1.3 J·kg^−1^·K^−1^ at the magnetic field change of *μ*_0_Δ*H* = 1 T (Figure 3b), which represents an increase in the order of magnitude compared to previously published HA nanomaterials, although the values of the magnetic entropy change of the nanowires prepared within this research do not exceed the magnetic entropy change of the most efficient magnetocaloric materials in their bulk form [66]. Nevertheless, the prepared nanowires might be used in small devices, employing their magnetocaloric effect for temperature changes in the nanoscale.

Later, it was shown that shape memory and magnetocaloric effect can be tuned by a properly selected chemical composition of bulk HAs. Either transition temperature, straining, or entropy change can be enhanced by using off-stoichiometric composition or by alloying with a small amount of transition metals [11,67].

Electrodeposition brings advantages in alloy production as it allows a proper chemical composition tuning, as well as enhancement of the miscibility of metals that are immiscible in the case of the classical arc-melting or induction-melting method [68,69]. This manuscript presents the production and basic structural and magnetic characterization of Ni_2_FeZ (Z = Ga, In, Sn) nanowires.

## 2. Materials and Methods

The fabrication of the nanowires within this research is based on a template-assisted electrochemical deposition. The electrodeposition was carried out in an electrochemical cell, consisting of the cylindrical platinum mesh at the cell’s walls as a counter electrode and a nanoporous AAO membrane with the diameter of the nanodimensional pores of *d* ≈ 60 nm, which also determined the diameter of the fabricated nanowires. The pores contain gold nanocontacts which serve as the working electrode located at the bottom of the AAO membrane. The electrochemical cell has been designed in a 3D modeling software in progression to achieve the most suitable design for quick and tunable electrodeposition of nanowires.

The electrochemical process depends on various degrees of freedom. Those include temperature, stirring, pH, deposition current density, electrodeposition time, and concentration of the individual metallic ions within the solution. Moreover, additives and their concentration variation can also be used to adjust or inhibit the co-electrodeposition of various metallic ions. Therefore, to obtain a Heusler-based composition of the prepared nanowires, the research was first focused on the optimization of the individual degrees of freedom and the search for suitable electrodeposition conditions for the preparation of HA nanowires.

In this article, the following nanowire samples were prepared to show the possibility of fabrication of various HA-based compositions in the nanoscale: Ni_66_Fe_21_Ga_13_, Ni_58_Fe_28_In_14_, and Ni_50_Fe_31_Sn_19_. The Ni_66_Fe_21_Ga_13_ nanowires were grown electrochemically from the following electrochemical bath: NiSO_4_ (10 mM), FeSO_4_ (3.2 mM), Ga_2_(SO_4_)_3_ (4 mM), Na_3_-Citrate (10 mM), H_3_BO_3_ (50 mM) and (NH_4_)_2_SO_4_ (10 mM). The pH of the electrolyte was optimized to the value of 3. The electrochemical deposition was carried out as a galvanostatic process with a current density of 5 mA·cm^−2^ for 1500 s. The Ni_58_Fe_28_In_14_ nanowires were grown electrochemically from the following electrochemical bath: NiSO_4_ (500 mM), FeSO_4_ (160 mM), In_2_(SO_4_)_3_ (50 mM), Na_3_-Citrate (500 mM), and H_3_BO_3_ (500 mM). The pH of the electrolyte was optimized to the value of 3. The electrochemical deposition was carried out as a galvanostatic process with a current density of 10 mA·cm^−2^ for 300 s. The Ni_50_Fe_31_Sn_19_ nanowires were grown electrochemically from the following electrochemical bath: NiSO_4_ (290 mM), FeSO_4_ (40 mM), Ga_2_(SO_4_)_3_ (40 mM), ascorbic acid (2 g·dm^−3^), sodium gluconate (120 g·dm^−3^) and H_3_BO_3_ (300 mM). pH value was not optimized. The electrochemical deposition was carried out as a galvanostatic process with a current density of 30 mA·cm^−2^ for 90 s. All the reagents were obtained from Merck (Darmstadt, Germany).

A MIRA3 TESCAN (TESCAN, Brno, Czech Republic) field-emission gun electron microscope was used to confirm the chemical composition of the fabricated nanowire samples and to study the nanowires’ morphology. Where applicable, the X-ray diffraction experiment was performed with a Rigaku D/Max II Rapid (RIGAKU Europe, Neu-Isenburg, Germany) X-ray diffractometer in a transmission geometry. The transmission electron microscopy and selected area electron diffraction were performed in a Fei Tecnai F20 field emission gun transmission electron microscope operated at 200 kV with a double-tilt specimen holder.

The magnetic and magnetocaloric properties of the prepared materials have been measured using a VSM (Vibrating Samples magnetometry), employing a SQUID (Superconducting Quantum Interference Device) measuring module within the Quantum Designs MPMS3 magnetometer (Quantum Design GmbH, Pfungstadt, Germany). The nanowires have been measured in an array inside the AAO membrane, aiming for a sufficient magnetic moment signal. The orientation of the nanowires within the array, with respect to the applied magnetic field during the measurement, is either parallel or perpendicular.

The distribution of the materials’ magnetic properties (coercivity and interaction field distributions), in the form of a First Order Reversal Curve (FORC) contour plot, was calculated using Equation (1) [70]:

(1) $$\begin{array}{c} \rho_{FORC}\left(H_{r,}H\right)=-\frac{1}{2}\frac{\partial^{2}M_{FORC}\left(H_{r},H\right)}{\partial H.\partial H_{r}}, \end{array}$$

where $\rho_{FORC}\left(H_{r,}H\right)$ is the FORC distribution at the given magnetic field *H* with the reversal field $H_{r,}$ from which the individual FORC measurements started. $M_{FORC}\left(H_{r},H\right)$ represents the measured magnetization at the given magnetic fields. The results are usually displayed in the field plane with the following coordinates (Equation (2)) [70,71]:

(2) $$\begin{array}{c} H_{C}=\frac{\left(H-H_{r}\right)}{2},\;H_{I\;}=\frac{\left(H+H_{r}\right)}{2}, \end{array}$$

where $H_{C}$ represents the coercive field and $H_{I}$ is the interaction field within the measured specimen. The results are depicted as a 2D contour plot with the $H_{C}$ and $H_{I}$ coordinates.

The FORC evaluation itself is predeceased by the measurement of the individual FORCs. At first, the positive saturating magnetic field (*H_S_*) is applied to the sample during the measurement. Then, a smaller value of the magnetic field is applied with the step of *H*_1_ = *H_S_* − *H_R_*, where *H_R_* is a reversal magnetic field step. Next, the sample is magnetically saturated again, and a field of the magnitude *H*_2_ = *H*_1_ − *H_R_*, is applied. The procedure is repeated until the negative magnetic saturation is achieved with the field steps of *H_n_* = *H*_*n*−__1_ − *H_R_* [70,71].

Temperature-First Order Reversal Curve (T-FORC) measurements consist of individual reversal curves measured within the hysteretic region of the *M*(*T*) dependence. During the cooling-TFORC analysis (which was used in this research), the sample is firstly heated above the transformation temperature, followed by a magnetization measurement during the cooling process. Then, the temperature was reversed to a temperature of *T_a_* = *T* − *T_R_*, where *T_R_* is the reversal temperature, and the cooling *M*(*T*) dependence is measured again. The temperature sweep rate was the same as with the *M*(*T*) dependence (3 K·min^−1^). The TFORC analysis consists of several individual measurements with equal steps of decreasing *T_R_* values. The *M_C_* (*T*, *T_R_*) values measured during the cooling TFORC analysis are evaluated according to (Equation (3)) [72,73,74]:

(3) $$\rho_{C,TFORC}\left(T,T_{r}\right)=-\frac{\partial^{2}M_{C}\left(T,T_{r}\right)}{\partial T\partial T_{r}},$$

where $\rho_{C,TFORC}\left(T,T_{r}\right)$ is the TFORC distribution of the cooling measurement. The TFORC distribution is then plotted as a function of the temperature hysteron width ($T_{H}$) and its central position ($T_{U}$) according to (Equation (4)) [72,73,74]:

(4) $$T_{h}=\frac{\left|T-T_{r}\right|}{2},\;T_{u}=\frac{T+T_{r}}{2}$$

The magnetocaloric behavior within this research was studied using an indirect approach, where the magnetization is measured under isothermal conditions at different temperatures. Before each isothermal measurement, the sample was heated to the maximum temperature of the MPMS3 system to ensure the presence of the high-temperature phase in the nanowires at the beginning of each measurement, where applicable. After the measurement of the isothermal magnetization dependence, the magnetic contribution to the overall entropy change was calculated according to Maxwell’s relation (Equation (5)) [75]:

(5) $$\Delta S_{M}=\mu_{0}\overset{H}{\underset{0}{\int }}{\left(\frac{\partial M}{\partial T}\right)}_{H}dH$$

Analysis of magnetic properties of the Ni-Fe-Sn set of nanowires was obtained employing a MicroSense EZ VSM system, due to a higher experimental temperature range, to study the second-order phase transformation-related magnetocaloric response of these nanowires.

## 3. Results and Discussion

It has been shown previously [64] that off-stoichiometric Ni-Fe-Ga-based nanowires show shape-memory behavior. However, the composition was far from stoichiometric [64]. Therefore, a new series of Ni-Fe-Ga nanowires was prepared, aiming to increase the Ga content in the resulting Ni-Fe-Ga nanowires to approach the stoichiometric composition. These nanowires show the chemical composition of Ni_66_Fe_21_Ga_13_.

### 3.1. Electrodeposited Ni_66_Fe_21_Ga_13_ Nanowires

**Figure 4:**
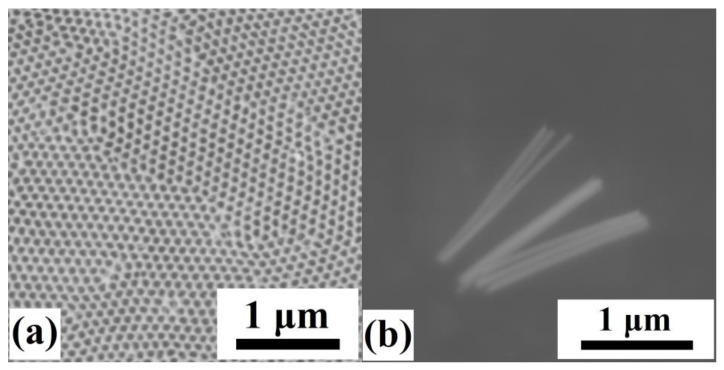
(**a**) AAO membrane that was used to fabricate the Ni_66_Fe_21_Ga_13_ nanowires; (**b**) SEM image of the nanowires after their release from the AAO membrane.

Nanowires that resulted from electrodeposition using the optimized electrochemical bath are shown in Figure 4b. Their length is approximately 1.5 μm, and the diameter is 60 nm, determined by the diameter of the individual nanopores of the AAO membrane (Figure 4a).

**Figure 5:**
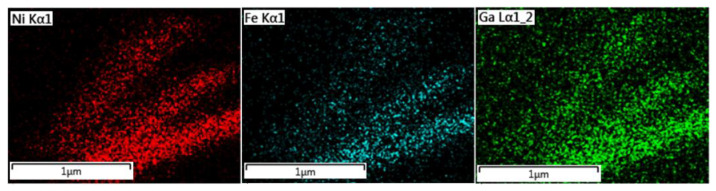
EDS maps of the Ni_66_Fe_21_Ga_13_ nanowires show a homogeneous distribution of the individual elements within the nanowire bodies (The EDS maps were collected from the spot shown in Figure 4b).

Scanning electron microscope analysis on free-standing nanowires confirmed the homogeneity of the distribution of the individual elements (Ni, Fe, Ga) within the nanowires’ chemical composition. This analysis was performed through Energy-Dispersive Spectroscopy (EDS) mapping (Figure 5). The results show that the individual elements within the nanowires are distributed homogeneously.

Structural analysis through X-ray diffraction (XRD) has been performed on an array of the prepared nanowires within the AAO template, which also contains the gold nanocontacts, from which the nanowires are grown.

**Figure 6:**
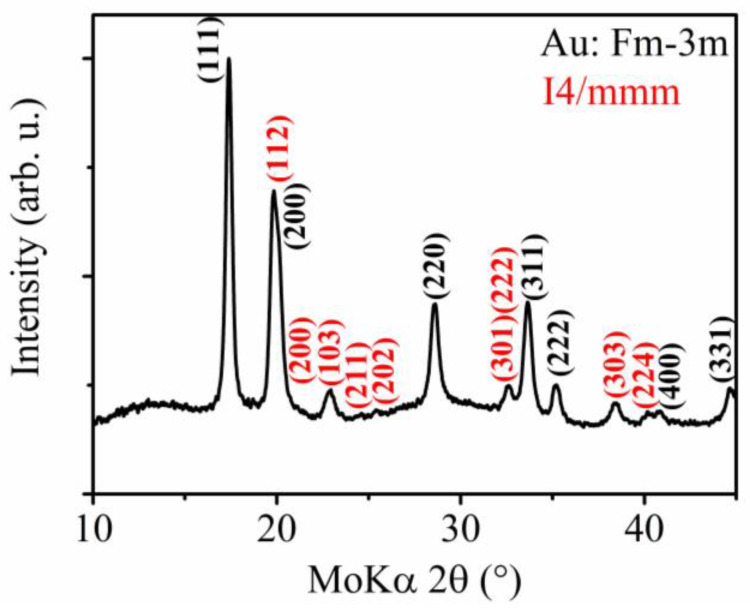
XRD analysis of the Ni_66_Fe_21_Ga_13_ nanowires inside the AAO template which also contains the gold nanocontacts in the individual nanodimensional pores.

**Figure 7:**
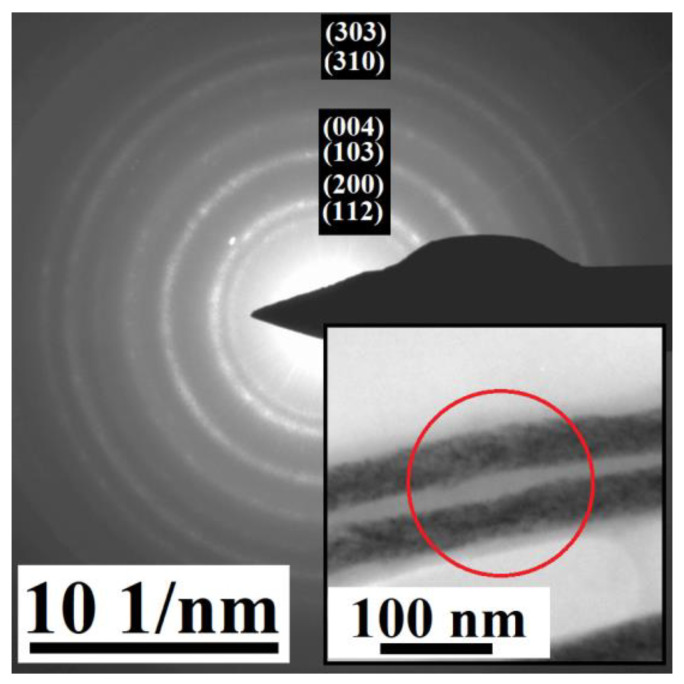
Electron diffraction pattern of the Ni_66_Fe_21_Ga_13_ nanowires with the most prominent *I4/mmm* diffraction maxima and the inset showing one of the spots (red circle) that were used to collect the 2D electron diffraction patterns (polycrystalline diffraction rings) during the Selected Area Electron Diffraction (SAED) experiment.

The XRD (Figure 6) reveals the presence of a tetragonal lattice with the space group *I4/mmm*. The lattice parameters are *a* = 3.85 Å and *c* = 6.11 Å. This space group may be ascribed to a Ni-Fe-Ga martensitic phase that has also been observed previously [76]. However, the pattern also shows the presence of a gold *Fm-3m* structure with a lattice parameter *a* = 4.06 Å, arising from the gold nanocontacts from the AAO membrane. Therefore, the structure was confirmed through an electron diffraction experiment on individual nanowires, removed from the AAO template to obtain the pure nanowires’ signal (Figure 7). The analysis shows polycrystalline nanowires’ structure resulting in continuous rings within the electron diffraction pattern. The electron diffraction was performed on several spots of the whole sample holder, showing the same pattern. This points to the structure of the nanowires being homogeneous among the whole analyzed part of the nanowires array.

The tetragonal *I4/mmm* structure seems to be present within the individual nanowires, as well as in the averaged signal from an array of nanowires obtained during the XRD analysis. The comparison also shows that the gold signal overlaps some of the diffraction maxima of the XRD results.

**Figure 8:**
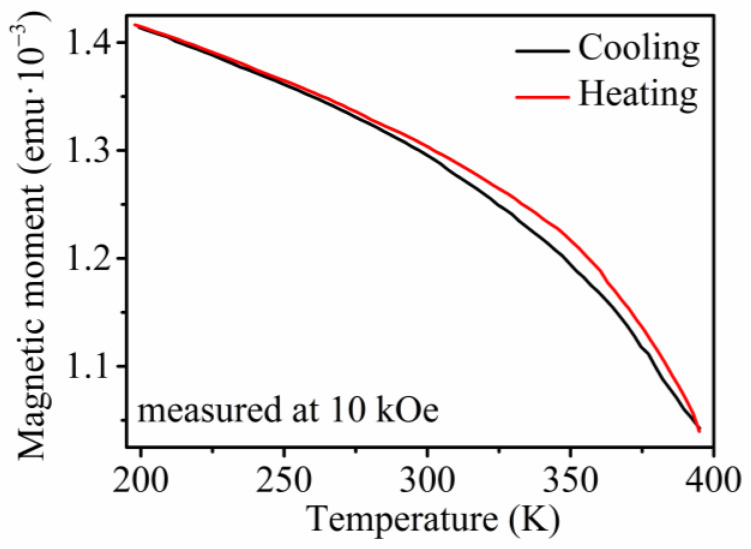
Temperature dependence of magnetization of the Ni_66_Fe_21_Ga_13_ nanowires showing a magnetization hysteresis above room temperature that might point to a phase transformation.

The confirmation of the nanowires’ composition, which approaches the HA X_2_YZ stoichiometry and the structural analysis, was followed by the search for their functional behavior. As described, the Ni_2_FeGa HA in a bulk macroscopic form shows a martensitic transformation capability, ensuring magnetocaloric and shape-memory behavior. Such behavior of the prepared nanowires can be detected through the analysis of the nanowires’ magnetic properties. This analysis started with the measurement of the temperature dependence of magnetization—*M*(*T*). The *M*(*T*) analysis (Figure 8) of the Ni_66_Fe_21_Ga_13_ nanowires shows a change in the nanowires’ magnetization, which occurs at the temperature of ≈370 K. During this analysis, the sample is first heated up to 395 K. Afterwards, the magnetic field of 10 kOe is applied for the *M*(*T*) analysis. The hysteretic behavior of magnetization between 250 and 395K points to the presence of a phase transformation. Therefore, further analysis of the Ni_66_Fe_21_Ga_13_ nanowires has been performed. The broad temperature range might be caused by the cascading nature of the phase transition within an array of interacting nanowires, as was discussed in the previous research [65] as well as by the local inhomogeneities within the nanowires’ composition in the entire AAO membrane since it was shown that the phase transformation temperature varies with small compositional changes in the previous research on bulk or micrometer-scale functional HAs [67].

**Figure 9:**
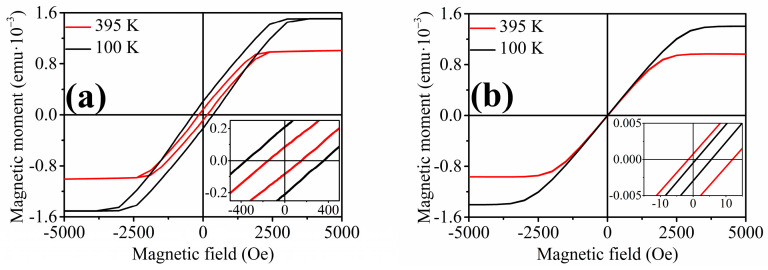
Hysteresis loops of the Ni_66_Fe_21_Ga_13_ nanowires measured in the parallel (**a**) and perpendicular (**b**) direction of the applied magnetic field at the temperatures of 100 K and 395 K.

Measurement of hysteresis loops offers the first insight into magnetization behavior at various temperatures. The hysteresis loops of the nanowires at different temperatures point to a strong interaction of the nanowires within the array since the saturating magnetic field acquires a high value of 5 kOe. Measurements of the hysteresis loops at temperatures of 100 K and 395 K show different characteristic features of the Ni-Fe-Ga nanowires (Figure 9). The saturation magnetic field of these nanowires changes from 3000 Oe at the temperature of 100 K to a value of 2500 Oe at the temperature of 395 K. Moreover, the coercivity changes from 353 Oe at the temperature of 100 K to the value of 150 Oe at 395 K. Such a change in the characteristic features of the hysteresis loop at different temperatures might also point to the desired functional behavior. Hysteresis loops were also measured in the perpendicular direction of the applied magnetic field with respect to the longitudinal axes of the nanowires. Here, the coercivity values drop below 10 Oe, which points to the easy magnetization direction being almost parallel to the longitudinal axes of the nanowires.

Although the hysteresis loops offer a tentative description of the magnetization process of a multi-domain material, more information can be obtained from the FORC and TFORC analysis. FORC analysis of an array of nanowires usually exhibits a two-branch (“T-shape”) structure [77]. One of the branches spreads along the *H_I_* axis and intersects the *H_I_* = 0 Oe region of the FORC distribution contour plot. The second branch lies in the *H_I_* = 0 Oe region along the *H_C_* axis and represents a distribution of the Preisach hysterons with a minimal interaction field but a notable coercive field distribution [77].

**Figure 10:**
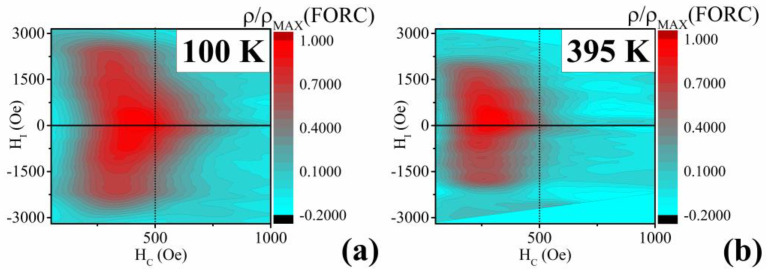
FORC analysis of the Ni_66_Fe_21_Ga_13_ nanowires at 100 K (**a**) and 395 K (**b**) the scale is the same for both temperatures, to emphasize the different FORC distribution.

The analyses have been performed at temperatures of 100 K and 395 K (Figure 10). At first, it can be seen that the nanowires show a rather homogeneous composition. This is apparent from the *ρ*(*FORC*) distribution being elongated along the *H_I_* axis, instead of the *H_C_*. Therefore, the whole set of nanowires shows a similar coercivity distribution within the whole studied part of the membrane, which might be due to the same composition of all the measured nanowires. Moreover, it has been shown that the diverging distribution shrinks with the decrease of outer dimensions [64]. As can be seen in Figure 10b, the elongation of the FORC distribution of the Ni-Fe-Ga nanowires decreases at the temperature of 395 K. This points to shape changes that may occur in the nanowires when they transform into a more symmetrical phase at high temperatures.

Next, the distributions show how the interaction of the nanowires varies at various temperatures. At 100 K, the *ρ*(*FORC*) values reach the maximum at the interaction field values of 0 Oe, while at 395 K, the maximum is reached at the values of *H_I_* = 150 Oe. This indicates a different distribution occurring at high temperatures, confirming the austenitic phase evolution.

The maximum of the *ρ*(*FORC*) values occurs at different *H_I_* for 100 K and 395 K measurements. At 100 K, the values start to increase at the coercive field of 100 Oe, and the distribution diminishes at *H_C_* = 700 Oe. At the high temperature, the whole distribution occurs between the *H_C_* values of 50 Oe and 500 Oe. Such behavior can be ascribed to a diminishing of the low-temperature phase at some point within the temperature range of 100 K and 395 K.

**Figure 11:**
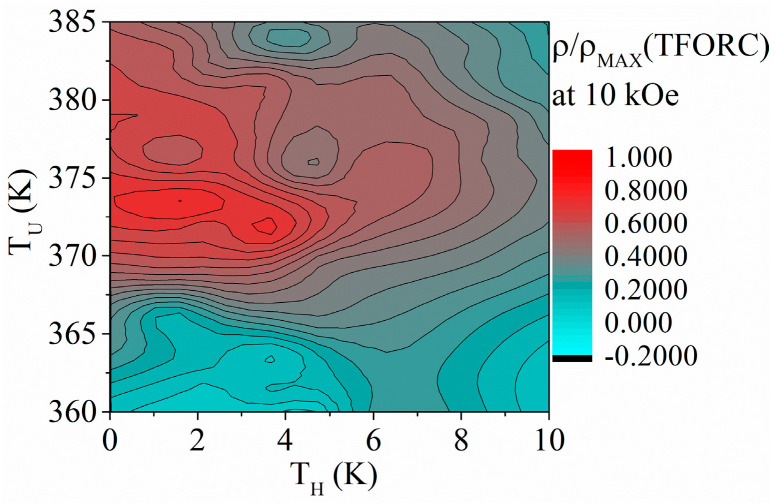
TFORC analysis of the Ni_66_Fe_21_Ga_13_ nanowires.

To confirm the proposed changes in the nanowires’ magnetic behavior, a TFORC analysis was performed. Here, the TFORC distribution is studied in the temperature plane, where the *ρ*(*TFORC*) values are shown in a 2D contour plot as a function of the temperature hysteresis (*T_H_* on the X-axis) and temperature of the phase transformation (*T_U_* on the Y axis) (Figure 11). To prevent the magnetization process (domain wall movement and magnetization vector rotation) of the nanowires during the TFORC analysis, it was performed at the applied magnetic field of 10 kOe. This value is well above the saturation field, and all the nanowires are magnetically saturated. Therefore, the only changes that can be tracked arise from the phase transformation in the case of a material that is capable of a martensitic transformation. Figure 11 shows the TFORC distribution of the Ni-Fe-Ga nanowires. Here, the TFORC maximum can be seen at the transformation temperature of *T_U_* ≈ 373 K, with the temperature hysteresis distribution that ranges from 0 K to approx. 5 K. These values correspond to the measurement of the temperature dependence of magnetization.

Since the functional behavior of these nanowires might arise from their proposed martensitic transformation capability, it is possible to study their magnetocaloric response using the indirect method, evaluating the changes in the magnetic entropy of the nanowires array.

**Figure 12:**
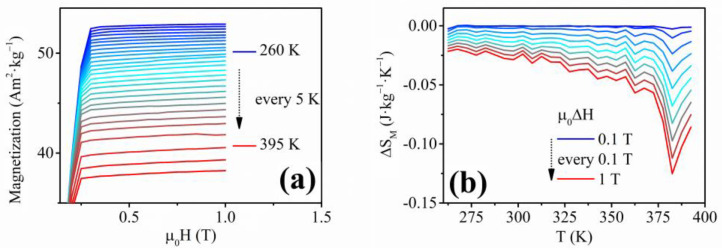
(**a**) Isothermal magnetization measurements of the Ni_66_Fe_21_Ga_13_ nanowires; (**b**) ΔS_M_ values calculated for the Ni_66_Fe_21_Ga_13_ nanowires.

To study the magnetocaloric behavior, the ΔS_M_ values were calculated from the isothermal magnetization measurements (Figure 12a). This figure shows the magnetic entropy change at various values of the applied magnetic field (Figure 12b). First, the magnetic entropy change of the Ni-Fe-Ga nanowires increases slowly, showing a typical ferromagnetic behavior. This can be seen as a slow increase of the Δ*S_M_* values as the temperature increases towards the Curie point. At the temperature of 360 K, a sharp peak begins to appear with the maximum at ≈380 K and the corresponding magnetic entropy change of |Δ*S_M_*| ≈ 0.12 J·kg^−1^·K^−1^ at the magnetic field change of *μ*_0_Δ*H* = 1 T, which is usually ascribed to a first-order phase transformation—martensitic transformation. A broad peak of magnetic entropy change also occurs as the material approaches the Curie temperature. However, the nanowires’ Curie temperature is above the experimental temperature range. Therefore, corresponding to the previous analysis, the first-order phase transformation begins to occur at the temperature of ≈375 K. As was mentioned earlier, around this temperature, the magnetic entropy change acquires the maximum values. Typically, the magnetic entropy change of a nanowire-shaped material shows the values of |Δ*S_M_*| ≈ 1 J·kg^−1^·K^−1^ at various temperatures and the change of magnetic field of up to *μ*_0_Δ*H* = 5 T [34,65,78]. On the other hand, these materials include expensive metals or perovskite materials, which have energy- and time-consuming fabrication procedures [34,65,78]. To avoid using expensive materials, a nanodimensional HA can be used repeatedly, despite the lower magnetocaloric response. A down-sized Ni-Fe-Ga HA in the form of a microwire shows a magnetic entropy change of up to 1 J·kg^−1^·K^−1^ due to the first-order phase transformation at the magnetic field change of *μ*_0_Δ*H* = 2 T [75]. The fabricated nanowires in this research show a lower magnetic entropy change compared to the mentioned materials, but it occurs at a much lower change in the magnetic field. Therefore, the efficiency of the magnetocaloric effect is higher due to an easier magnetic saturation ensured by the shape anisotropy, making the nanowires suitable for magnetocaloric applications in the nanoscale [34,65].

The maximum values of the Δ*S_M_* for Ni-Fe-Ga nanowires samples exceed the maximum entropy change of Ni–Mn–Ga nanowires published previously and produced by electrospinning (≈0.15 J·kg^−1^·K^−1^ at the higher applied magnetic field change of 2 T) [34], despite the superiority of the Ni-Mn-Ga HA regarding the magnetocaloric effect efficiency. Since the Ni–Mn–Ga HA nanowires prepared by the electrospinning method exhibit a random orientation, the applied magnetic field is perpendicular to a considerable number of nanowires. Hence, a higher applied magnetic field is necessary to saturate all the nanowires, which results in a lower magnetocaloric effect efficiency compared to the presented electrodeposited Ni-Fe-Ga nanowires. Therefore, the magnetocaloric behavior might result in a higher value of the magnetic entropy change at a lower applied magnetic field due to the homogeneous composition and the parallel alignment of the electrodeposited nanowires within this research.

### 3.2. Ni_58_Fe_28_In_14_ Nanowires

Current applications research regarding HAs also focuses on the tuning of their functional behavior, which might be achieved through a complete substitution of the individual constituents of the original HAs. Such an approach was also chosen during the fabrication of Ni-Fe-In nanowires, where the Ga^3+^ ions were replaced by In^3+^ in the solution that was used to prepare the Ni-Fe-Ga nanowires within this research. The electrodeposition approach is particularly suitable during the co-electrodeposition of the Fe^2+^ and In^3+^ ions since the pure metals of Fe and In are nearly immiscible within the standard high-temperature alloying process. Such insufficiency within alloy preparation can be solved by electrodeposition [69].

**Figure 13:**
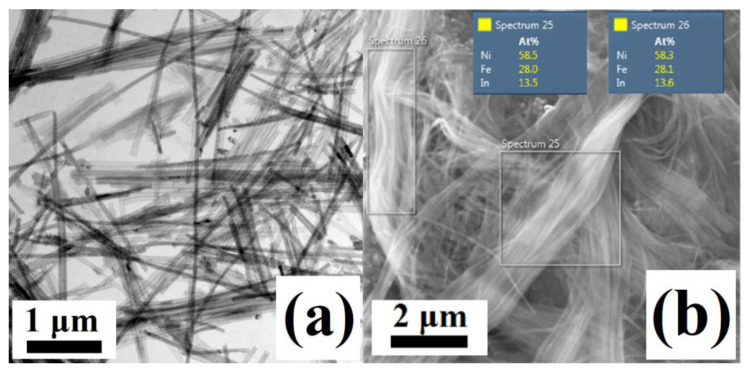
(**a**) TEM bright-field image of the electrodeposited Ni_58_Fe_28_In_14_, (**b**) SEM/EDS analysis of the Ni_58_Fe_28_In_14_ nanowires showing some of the areas that were chosen for the composition determination.

**Figure 14:**
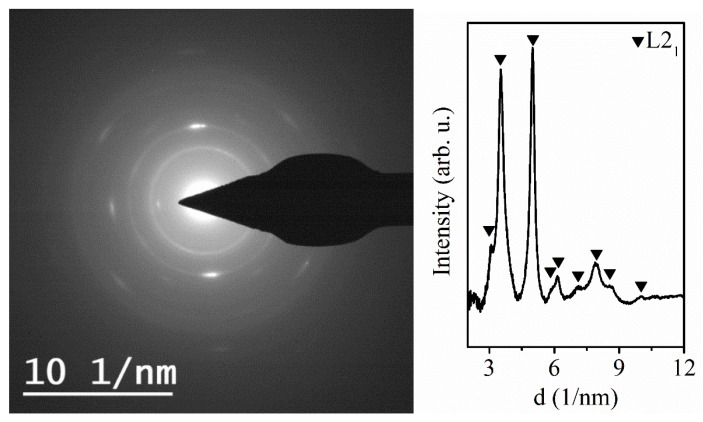
(**Left**) 2D electron diffraction pattern of the fabricated Ni_58_Fe_28_In_14_ nanowires, (**Right**) Integrated 2D pattern showing the contribution of the L2_1_ cubic phase to the nanowires’ composition.

The Ni_58_Fe_28_In_14_ nanowires show a length of approx. 10 μm, and the average chemical composition of Ni_58_Fe_28_In_14_ (Figure 13). Since the fabricated Ni_58_Fe_28_In_14_ nanowires provided no sufficient XRD signal, their structure was analyzed using the electron diffraction (SAED method) of the individual nanowires using the TEM (Figure 14). The obtained structural features can only be ascribed to a cubic *L2*_1_ lattice (*Fm-3m*) with a lattice constant of *a* ≈ 5.62 Å.

**Figure 15:**
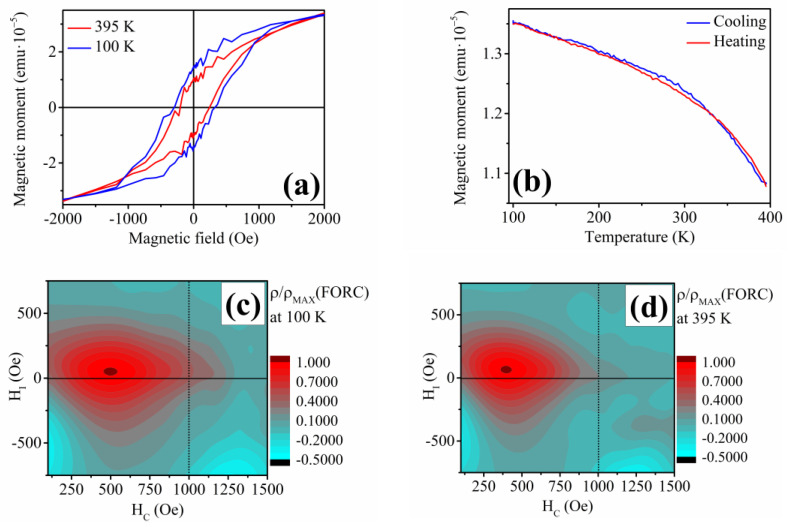
(**a**) Hysteresis loops of the Ni_58_Fe_28_In_14_ nanowires at 395 K showing negligent changes with temperature; (**b**) Low-field temperature dependence of magnetization of the Ni_58_Fe_28_In_14_ nanowires; (**c**) FORC analysis of the Ni_58_Fe_28_In_14_ at 100 K; (**d**) FORC analysis of the Ni_58_Fe_28_In_14_ nanowires at 395 K.

The fabricated Ni-Fe-In nanowires with the ordered HA structure show a weak magnetic moment signal and no significant changes in their magnetic behavior can be seen from the low-field temperature dependence of magnetization and hysteresis loops (Figure 15a,b). The temperature changes are accompanied by a slight change in the saturation magnetic field, together with the thermal decrease of the nanowires’ coercivity. The distribution of the magnetization properties (interaction field *H_I_* and coercivity *H_C_*) also shows a decrease in the area that might be ascribed to the change in the materials’ temperature. Nevertheless, the FORC contour plot shows the same *H_I_* distribution for both temperatures (Figure 15c,d), pointing to a lack of functional behavior.

### 3.3. Ni_50_Fe_31_Sn_19_ Nanowires

**Figure 16:**
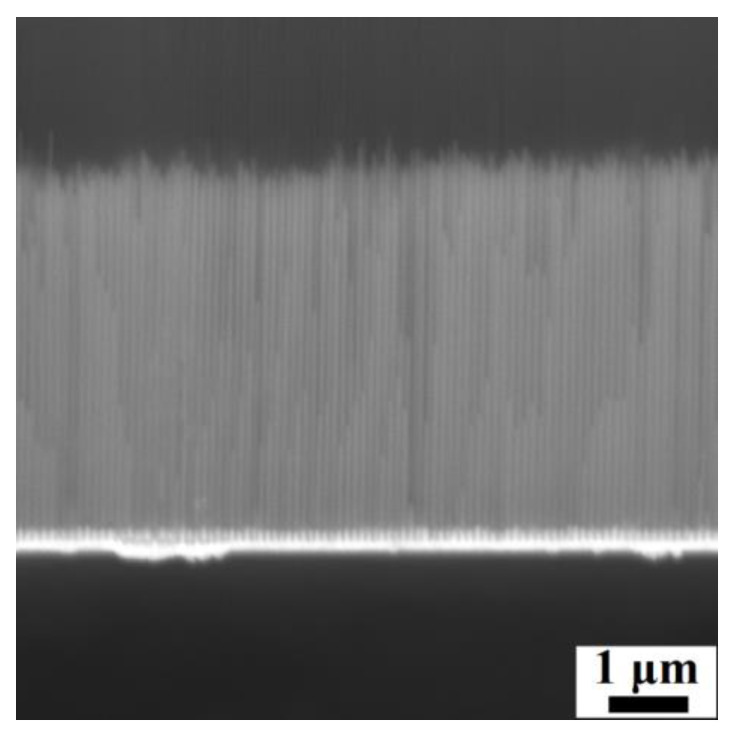
Fabricated Ni-Fe-Sn nanowires inside the AAO membrane.

**Figure 17:**
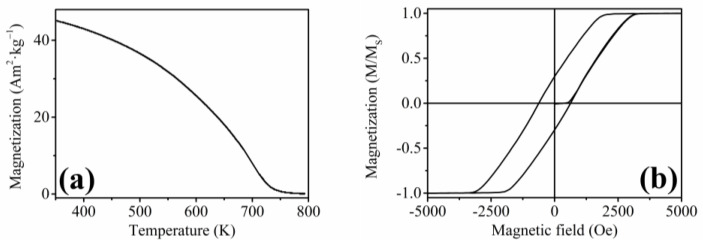
(**a**) *M*(*T*) dependence of the electrodeposited Ni-Fe-Sn nanowires above room temperature, showing the Curie point of approximately 750 K; (**b**) Hysteresis loop of the Ni-Fe-Sn nanowires with the coercivity of up to 750 Oe and magnetic saturation visible at ≈3.5 kOe.

The Ni-Fe-Sn nanowires show a length of approx. 5 μm (Figure 16). The functional behavior was initially studied through *M*(*T*) analysis above room temperature (Figure 17a), which showed no significant changes that could be ascribed to a phase transition. The structural analysis of the Ni-Fe-Sn nanowires was not performed since no significant evidence was observed in the magnetic measurements, which would indirectly indicate structural transformation. The hysteresis loop, measured at room temperature, shows a coercivity of ≈ 700 Oe and a saturation field of 3.3 kOe, which points to a strong interaction among the neighboring nanowires, which results in a leaning of the nanowires’ hysteresis loop toward higher saturation magnetic field (Figure 17b) [79].

**Figure 18:**
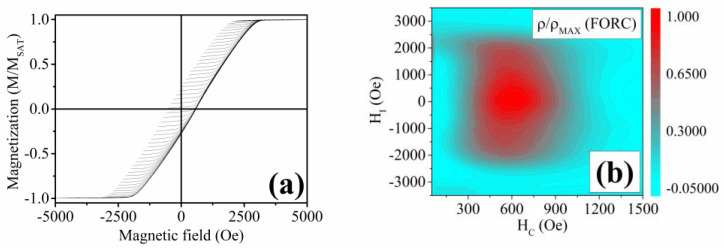
(**a**) Measurements of the individual FORCs of the Ni-Fe-Sn nanowires at room temperature; (**b**) Evaluated contour plot of the Ni-Fe-Sn nanowires’ ρ(FORC) distribution.

The FORC analysis was performed to determine the interaction and coercivity field distributions of the Ni-Fe-Sn nanowires (Figure 18a). It can be seen that the majority of the nanowires exhibit a coercive field between 450 and 700 Oe, which might represent a small inhomogeneity of the electrodeposited Ni-Fe-Sn nanowires (Figure 18b). The interaction field distribution of ±3000 Oe shows that the array of Ni-Fe-Sn nanowires is a highly interacting and multi-domain material (Figure 18b), as has been ascribed to nanowires in the published research [77].

**Figure 19:**
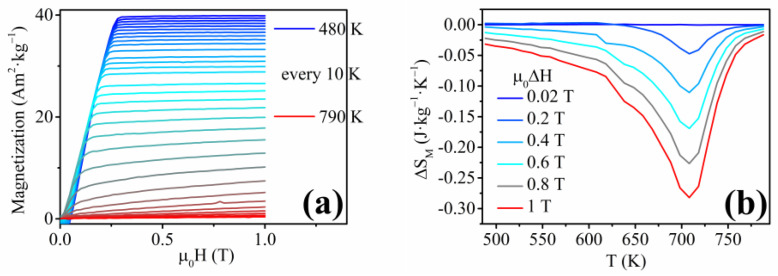
(**a**) Isothermal magnetization measurements above room temperature showing a gradual decrease of *M_S_* of the Ni-Fe-Sn nanowires; (**b**) Magnetic entropy change of the Ni-Fe-Sn nanowires pointing to a 2nd order phase transformation due to a broad peak of the Δ*S_M_*.

The lack of shape-memory functionality corresponds to the previous analysis by Frolova et. al, where the bulk Ni-Fe-Sn alloys did not exhibit the presence of the ordered *L2*_1_ cubic phase of the HAs [80]. The Ni_2_FeSn alloys in the bulk form showed the presence of a hexagonal structure with the space group *P6*_3_*/mmc* [80]. Nevertheless, the unique Ni-Fe-Sn nanowires were analyzed to obtain their magnetocaloric response in relation to the second-order phase transition (Curie point). The indirect approach was chosen to allow a comparison of the previously published research with the novel nanowire materials. The measurements of the isothermal magnetization with decreasing temperature are shown in Figure 19a. The *T_C_* of the Ni-Fe-Sn nanowires is approximately 750 K (from *M*(*T*) measurement), and the individual *M*(*H*)*_T_* measurements correspond to the *M*(*T*) analysis, showing a gradual decrease of *M_S_* with temperature until *T_C_*. The magnetic entropy change (Figure 19b) has a peak at the Curie temperature with the maximum magnetic entropy change of Δ*S_M_*(*max*) ≈ 0.25 J·kg^−1^·K^−1^ at the magnetic field change of *μ*_0_Δ*H* = 1 T.

## 4. Conclusions

In this article, a summarization of the published Heusler alloy nanowires is followed by our attempt to fabricate a functional nanowire array focusing on a near-stoichiometric composition. The Ni-Fe-Ga nanowires show first-order phase transition behavior, which was confirmed by subsequent analysis of the structural, magnetic, and magnetocaloric properties. The magnetic entropy change during the magnetocaloric analysis is ≈0.12 J·kg^−1^·K^−1^ at a magnetic field change of *μ*_0_Δ*H* = 1 T and a temperature of ≈375 K, which approaches previously published Ni-Fe-Ga Heusler alloy materials. The suitability of the nanowire arrays for magnetocaloric applications was discussed since they show a moderate, but repeatable magnetocaloric response, enabling the nanowires for magnetocaloric applications in the nanoscale.

The following substitution of Ga in the Ni-Fe-Ga composition resulted in near-stoichiometric Ni-Fe-In nanowires fabricated for the first time, which show a weak magnetic moment signal and no detectable functional behavior, despite the presence of a single *L2*_1_ cubic phase. Nevertheless, these results point to the possibility of fabrication of an alloy from immiscible metals using the electrodeposition approach. Substitution of Ga by Sn resulted in nanowires with moderate magnetocaloric response (due to the 2nd order phase transformation—Curie temperature at 750 K) of ≈0.25 J·kg^−1^·K^−1^ at the magnetic field change of *μ*_0_Δ*H* = 1 T.

The fabrication process was based on electrodeposition, which requires optimization of multiple degrees of freedom (such as current density/deposition potential, pH, temperature, concentration of the individual constituents of the electrodeposition bath, etc.). On the other hand, electrodeposition provides flexibility regarding the composition of the prepared material. Moreover, once optimized, electrodeposition yields billions of nanowires in several minutes. It is also a scalable method, which is desirable for practical applications of fabricated materials. In the future, the small inhomogeneities of the nanowires’ composition can be improved by the introduction of a new degree of freedom within the deposition process (such as stirring combined with a pulsed/AC electrodeposition). Nevertheless, the discussed results and the fabrication method of the Ni_66_Fe_21_Ga_13_, Ni_58_Fe_28_In_14_, and Ni_50_Fe_31_Sn_19_ nanowires offer the possibility of research on Heusler alloy nanowires with novel compositions that show functional behavior.

## Figures and Tables

**Table 1:** Chronological evolution of HA nanowires research; summary of fabricated nanowires with the fabrication method and aim of the cited HA research.

Composition	Research Aim	Fabrication	Ref.
Fe_3_Si	Binary HA nanowires	crystal transformation	2010 [32]
Co_2_FeAl	High T_C_, diameter < 500 nm	electrospinning	2012 [33]
Co_2_FeGa	Electron tomography on HA nanowires	Annealing in SBA-15 template	2014 [35]
Co_2_FeGa	Diameter below 7 nm	Annealing in SBA-15 template	2016 [36]
GaAs-Fe_3_Si	Semiconductor–Ferromagnet Core–Shell Nanowires	Molecular beam epitaxy	2017 [62]
Co_2_NiGa	Composition tuning through deposition potential	DC Electrodeposition	2017 [52]
Co_2_FeSn	Easy axis perpendicular to the nanowire axis	DC Electrodeposition	2018 [55]
Co_2_FeGa-SiO_2_	Diameter 4–7 nm, length below 200 nm	Chemical approach in SBA-15	2018 [36]
Co_2_Mn_0.5_Fe_0.5_Sn	100% spin-polarization, L2_1_ structure	AC Electrodeposition	2018 [54]
Fe_2_CoSn	Spintronic material due to suitable transport properties	AC Electrodeposition	2018 [53]
Co_2_FeIn	High spin polarization, suitable for spintronics	DC electrodeposition	2018 [43]
Co_2_MnSn	Easy axis parallel to the nanowire axis	AC Electrodeposition	2019 [57]
Co_2_MnAl	Composition tuning through deposition parameters	DC electrodeposition	2019 [58]
Ni-Mn-Ga	Diameter of ≈200 nm	DC electrodeposition	2020 [59]
Co_2_FeSn	100% spin-polarization	DC electrodeposition	2020 [56]
Ni_2_MnGa	2nd order phase transformation MCE	electrospinning	2020 [34]
Ni_2_FeGa	Electrodeposited nanowires and nanotubes	DC electrodeposition	2021 [61]
Co_2_FeGa	Magnetoresistance properties of HA nanowires	Template assisted electrodeposition	2022 [60]
Fe_2_MnGa	Magnetic properties of novel HA nanowires	DC electrodeposition	2022 [63]
Ni-Fe-Ga	FORC + TFORC analysis of functional nanowires	DC electrodeposition	2022 [64]
Co_2_MnAl, Co_2_CrAl and Co_2_TiAl	Growth of nanowires and nanotubes	Magnetron Sputtering in AAO template	2022 [38]
Ni_2_MnGa	Magnetocaloric effect due to first-order phase transformation	DC electrodeposition	2023 [65]

## Data Availability

The raw data supporting the conclusions of this article will be made available by the authors on request.
